# 

**Published:** 1982

**Authors:** 


					vv/ im C >*
-i % 3 M fwmmWWWfWri -
NATIONAL^.
LIBRARY ^
S I 1 FES 1983 J3P
1 OF
ft
JANUARY/APRIL 1982
VOLUME 97 i/ii
NUMBER 361/362

				

